# Hepatorenal syndrome: the 8^th ^international consensus conference of the Acute Dialysis Quality Initiative (ADQI) Group

**DOI:** 10.1186/cc11188

**Published:** 2012-02-09

**Authors:** Mitra K Nadim, John A Kellum, Andrew Davenport, Florence Wong, Connie Davis, Neesh Pannu, Ashita Tolwani, Rinaldo Bellomo, Yuri S Genyk

**Affiliations:** 1 Department of Medicine, University of Southern California, 1520 San Pablo Street, Suite 4300, Los Angeles, CA, 90033 USA; 2 Department of Critical Care Medicine, University of Pittsburgh, Room 608, Scaife Hall, 3550 Terrace Street, Pittsburgh, PA 15261 USA; 3 Department of Medicine, University College London Medical School, Rowland Hill Street, London NW3 2PF UK; 4 Department of Medicine, University of Toronto, 9th Floor Rm. 9N983, 200 Elizabeth Street, Toronto, ON M5G 2C4 Canada; 5 Department of Medicine, University of Washington, Box 356174, 1959 NE Pacific Street, Seattle, WA 98195, USA; 6 Department of Medicine, University of Alberta, 11-107 csb 8440 112 Street, Edmonton, Canada T6G 2G3, AB, Canada; 7 Department of Medicine, University of Alabama, ZRB 605, 1530 3rd Ave. S, Birmingham, AL 35294-0007 USA; 8 Australian and New Zealand Intensive Care Research Centre, School of Public Health and Preventive Medicine, Monash University, Alfred Centre, Commercial Rd, Prahran, Melbourne, Victoria 3181, Australia; 9 Department of Surgery, University of Southern California, 1510 San Pablo Street, Suite 200, Los Angeles, CA, 90033 USA

**Keywords:** hepatorenal syndrome, cirrhosis, acute kidney injury, ADQI, RIFLE

## Abstract

**Introduction:**

Renal dysfunction is a common complication in patients with end-stage cirrhosis. Since the original publication of the definition and diagnostic criteria for the hepatorenal syndrome (HRS), there have been major advances in our understanding of its pathogenesis. The prognosis of patients with cirrhosis who develop HRS remains poor, with a median survival without liver transplantation of less than six months. However, a number of pharmacological and other therapeutic strategies have now become available which offer the ability to prevent or treat renal dysfunction more effectively in this setting. Accordingly, we sought to review the available evidence, make recommendations and delineate key questions for future studies.

**Methods:**

We undertook a systematic review of the literature using Medline, PubMed and Web of Science, data provided by the Scientific Registry of Transplant Recipients and the bibliographies of key reviews. We determined a list of key questions and convened a two-day consensus conference to develop summary statements via a series of alternating breakout and plenary sessions. In these sessions, we identified supporting evidence and generated recommendations and/or directions for future research.

**Results:**

Of the 30 questions considered, we found inadequate evidence for the majority of questions and our recommendations were mainly based on expert opinion. There was insufficient evidence to grade three questions, but we were able to develop a consensus definition for acute kidney injury in patients with cirrhosis and provide consensus recommendations for future investigations to address key areas of uncertainty.

**Conclusions:**

Despite a paucity of sufficiently powered prospectively randomized trials, we were able to establish an evidence-based appraisal of this field and develop a set of consensus recommendations to standardize care and direct further research for patients with cirrhosis and renal dysfunction.

## Introduction

Hepatorenal syndrome (HRS) is a unique form of kidney injury resulting from renal vasoconstriction in the setting of systemic and splanchnic arterial vasodilatation in patients with advanced cirrhosis. HRS is typically subdivided into two types: type-1 in which there is a rapid deterioration in kidney function with the serum creatinine (Scr) increasing by more than 100% from baseline to greater than 2.5 mg/dl within a two-week period, whereas type-2 HRS occurs in patients with refractory ascites with either a steady but moderate degree of functional renal failure (≥ 1.5 mg/dl) or a deterioration in kidney function that does not fulfill the criteria for HRS type-1 [1]. In patients with advanced cirrhosis, HRS is reported to occur in 18% within one year of diagnosis and up to 40% at five years [2]. Untreated, median survival is two weeks for patients with type-1 HRS and four to six months in patients with type-2 HRS [3]. However, many patients with lesser degrees of renal impairment in the setting of cirrhosis do not meet the precise definitions of HRS and, yet, have the same pathophysiological basis to their renal impairment and, therefore, could potentially benefit from therapies developed for HRS. A consensus conference under the auspices of the Acute Dialysis Quality Initiative (ADQI) was held in 2010. The purpose of this consensus conference was to review the literature on renal impairment in cirrhosis and to create the basis for a definition and classification system of all forms of renal impairment including HRS, from there to develop a broader understanding of its epidemiology, review current knowledge in the field of prevention and treatment, and develop the framework for a research agenda in relation to this condition.

## Material and methods

### ADQI process

ADQI is an ongoing process that seeks to produce evidence-based recommendations for the prevention and management of acute kidney injury (AKI) and on different issues concerning acute dialysis. It represents a non-profit association with an elected rotating board. ADQI conducts systematic reviews of the literature and provides expert-based statements and interpretation of current knowledge for use by clinicians according to professional judgment. The ADQI methods comprise (1) systemic search for evidence with review and evaluation of the available literature, (2) establishment of clinical and physiologic outcomes as well as measures to be used for comparison of different treatments, (3) description of the current practice and the rationale for use of current techniques, and (4) analysis of areas in which evidence is lacking and future research is required.

Prior to the conference, the organizing committee of ADQI VIII identified five topics relevant to the field of HRS (Table 1). We selected these topics with a set of key questions based on (1) prevalence of the associated clinical problem, (2) known variation in clinical practice, (3) availability of scientific evidence, (4) potential importance for clinical outcome, and (5) development of evidence-based medicine guidelines. For each topic, we outlined a preliminary set of key questions. We then assembled a diverse international panel representing multiple relevant disciplines (nephrology, hepatology, transplant surgery and critical care), from a variety of countries and scientific societies based on their expertise in AKI and HRS. Panelists were assigned to four to five person work groups, one of whom served as the group facilitator with each work group addressing one key topic.

**Table 1:** Topics covered and excluded in the Consensus Conference

Topics Covered	Topics Excluded
• Evaluation of renal function in patients with cirrhosis • Current definition and Classification of HRS • Pharmacologic treatment of HRS • Device management of HRS • Surgical and interventional management of HRS	• Indication for renal replacement initiation • Determinants for patient selection for extracorporeal system • Patient selection for liver transplantation • Post transplant immunosuppresion management

ADQI activities were divided into a pre-conference, conference and post-conference phase. During the pre-conference phase, topics were selected, and the work groups were assembled and assigned to specific topics. Each group identified a list of key questions, conducted a systematic literature search and generated a bibliography of key studies. During this stage, the scope of the conference was also defined and some topics were excluded (Table 1). We then conducted a 2 1/2 -day conference. Our consensus process relied on evidence where available and, in the absence of evidence, consensus expert opinion where possible. The quality of the overall evidence and the strength of recommendations were graded using the Grading of Recommendations Assessment, Development and Evaluation system (Table 2) [4-6]. For interventions that have been studied using randomized trials we pooled the results of various trials using the Mantel-Haenszel method for calculating the weighted summary odds ratio under the fixed effects model. We also calculated the heterogeneity statistic and incorporated it to calculate the summary odds ratio under the random effects model [7,8]. Both fixed and random effects models are reported.

**Table 2:** Grading evidence and recommendations (adapted from the GRADE system)

	Notes	Symbol
**Quality of Evidence**		
High	Large, high quality randomized control trials. We are confident that the true effect lies close to that of the estimate of the effect.	A
Moderate	Limited or conflicting data from randomized control trials. The true effect is likely to be close to the estimate of the effect, but there is a possibility that it is substantially different.	B
Low	Observational studies or very small randomized control trials. The true effect may be substantially different from the estimate of the effect.	C
Very low	Expert opinion. The estimate of effect is very uncertain, and often will be far from the truth.	D
**Grading Recommendation^a^**		
Strong 'We recommend'	Conditions for which there is evidence and/or general agreement that a given procedure or treatment is beneficial, useful and effective	1
Weak 'We suggest'	Conditions for which there is conflicting evidence and/or divergence of opinion about the usefulness/efficacy of a procedure or treatment	2

^a^'Not Graded' was used to provide guidance where the topic does not allow adequate application of evidence.

During the conference, work groups assembled in breakout sessions, as well as in plenary sessions where their findings were presented, debated and refined. In each breakout session, the work groups refined the key questions, identified the supporting evidence, and generated practice recommendations and/or directions for future research as appropriate. A series of summary statements was then developed during the breakout sessions and presented to the entire group, revising each statement as needed until a final version was agreed upon. Directives for future research were achieved by asking the participants to identify deficiencies in the literature, determine if more evidence was necessary and if so, to articulate general research questions. When possible, pertinent study design issues were also considered. Final reports were summarized into a final conference document by a writing committee.

### Systematic review of the literature

For each topic, the systematic review included the development of well specified research questions, literature searches, data extraction of primary studies and existing systematic reviews, tabulation of data, assessment of the quality of individual studies, and assessment of the overall quality of the literature and summary conclusions. Literature review was applied using key terms relevant to the topic and electronic reference libraries with the focus on human studies and limited to English language articles published between January 1960 and December 2009. Study eligibility was based on population, intervention, comparator, outcome, and study design relevant to each clinical question. Although nonrandomized studies were reviewed, the majority of the Work Group resources were devoted to review of randomized trials, as these were deemed to be most likely to provide data to support level 1 recommendations with very high- or high-quality (A or B) evidence. Exceptions were made for topics with sparse evidence. Each work group conducted literature searches related to their topic questions via MEDLINE, PubMed, data provided by the Scientific Registry of Transplant Recipients, Web of Science and the bibliographies of key reviews and of all articles that met the search criteria. Decisions to restrict the topics were made to focus the systematic reviews on those topics in which existing evidence was thought to be likely to provide support for the guideline.

### Evaluation of studies

A three-phase approach was used to construct the evidence-based recommendations. The phases included a systematic literature review of studies in HRS and AKI in patients with cirrhosis, a comprehensive appraisal of prior studies, and convening an expert panel to synthesize this information and develop consensus based recommendations. The quality of the overall evidence and the strength of recommendations were graded using the Grading of Recommendations Assessment, Development and Evaluation system (Table 2) [4-6]. There were four categories for the quality of overall evidence, ranging from A to D. The strength of a recommendation was determined by the quality of the evidence and was graded Level 1, 2 or 'not graded". Recommendations were "Not Graded" if they were not based on systematic evidence and was used to provide guidance where the topic did not allow adequate application of evidence. Recommendation statements were developed from the systematic review, existing guidelines identified in the supplemental literature review, and expert opinion. Recommendations were developed by incorporating the best available evidence and expert opinion. Expert opinion was used when evidence in the literature did not exist to inform the decision. Recommendation statements were incorporated when they met one of two criteria: if there was strong literature-based evidence or if the expert panel voted that the recommendation was appropriate.

## Results and discussion

### I. Evaluation of renal function in patients with cirrhosis

#### 1. Serum creatinine measurements should be used to evaluate renal function in patients with advanced cirrhosis until more reliable methods of measuring renal function become generally available (1D)

**Rationale**: Serum creatinine (S_Cr_) measurement remains the most practical and widely accepted method for estimating renal function in clinical practice in patients with cirrhosis and is the basis of existing definitions of AKI. The prognostic impact of renal function in liver disease is reflected by the inclusion of S_Cr _in the Model for End-Stage Liver Disease (MELD) score, which is used to prioritize patients for liver transplantation [9]. However, in cirrhosis, S_Cr _is notoriously inaccurate in the diagnosis of renal dysfunction as it overestimates renal function due to decreased creatine production by the liver, protein calorie malnutrition, and muscle wasting [10-12]. Furthermore, the measurement of S_Cr _using the Jaffe method can be artificially lowered due to hyperbilirubinemia [13], or raised by cephalosporins [14] leading to variability in MELD scores [15]. Serum cystatin C has been suggested as a sensitive marker of renal function [16-22]; however, recent studies have shown that like S_Cr_, cystatin C is affected by age, gender, muscle mass and liver disease and overestimates renal function in patients with cirrhosis [17,23].

Glomerular filtration rate (GFR) is considered the best estimate of renal function although there is no universally accepted gold standard for its measurement. Clearance techniques using exogenous markers such as radiocontrast media, inulin or radioisotopes provide a more accurate measurement of GFR, but are labor intensive and expensive and appear to be susceptible to extra-renal clearance, overestimating GFR by as much as 20 mL/min/1.73 m^2 ^[24,25]. For patients with liver disease, particularly those with advanced cirrhosis, none of the exogenous clearance markers have been rigorously studied. When properly performed, timed urinary collection of creatinine overcomes some of these limitations [26,27], but due to increased renal tubular creatinine secretion, creatinine clearance overestimates GFR measured by inulin clearance by a mean of 13 mL/min/1.73 m^2 ^[27].

#### 2. GFR derived equations should be used cautiously for assessment of kidney function in cirrhosis since they tend to overestimate GFR (2D)

**Rationale: **The Cockcroft Gault [28] and Modified Diet in Renal Disease (MDRD) [29] equations are widely used to estimate GFR (eGFR) in the general population but consistently overestimate GFR in cirrhotic patients. The Cockcroft Gault equation is heavily influenced by weight as a reflection of lean body mass, which is not applicable to cirrhotic patients, in whom edema and ascites may account for a moderate and in some patients, even a substantial proportion of their weight [10,26,30,31]. Several retrospective evaluations of S_Cr_-based eGFR equations among liver transplant recipients suggest that the MDRD equations were best able to estimate GFR in comparison to radionucleotide GFR assessment; however, the precision of all GFR equations was poor [32,33]. Other eGFR equations such as the Chronic Kidney Disease Epidemiology Collaboration (CKD-EPI) and cystatin C based eGFR, have been proposed; however, like the MDRD equations, they have not been validated in patients with liver disease [34-38]. Therefore, we encourage very cautious use of these derived equations for the determination of GFR as they have not been validated in patients with liver disease.

Recommendations for future research:

• Formulate a specific equation for the calculation of GFR for patients with advanced cirrhosis by iohexol or inulin GFR determinations along with measurement of Scr and Cystatin C.

• Identify the role of ancillary renal testing such as diagnostic doppler ultrasound as epidemiological markers for patients with cirrhosis and renal dysfunction.

• Evaluate the value of renal injury biomarkers such as NGAL (neurtrophil gelatinase-associated lipocalin), IL-18 and KIM-1 (kidney-injury molecule) in the setting of AKI within the spectrum of hepatorenal disease.

### II. Definition and classification of renal impairment in cirrhosis

#### 1. Classify AKI in the setting of cirrhosis according to RIFLE criteria (Not Graded)

**Rationale: **In 1996, the International Ascites Club (IAC) proposed a definition and diagnostic criteria for HRS [39] which was later revised in 2007 (Table 3) [40]. However, the rigid cut off value of S_Cr _of ≥ 1.5 mg/dL has limited prompt management of patients with milder renal dysfunction. In 2004, the ADQI Workgroup developed a consensus definition and classification for AKI known as the RIFLE (Risk, Injury, Failure, Loss, End-Stage) criteria which stratified acute renal dysfunction into grades of increasing severity based on changes in S_Cr _and/or urine output [41]. Subsequently it was recognized that even smaller increases in S_Cr _(absolute increase in S_Cr _≥ 0.3 mg/dL) are associated with adverse outcome [42]. As a result, the criteria was modified in 2007 to broaden the definition of AKI (Table 4) [43].

**Table 3:** International Ascites Club (IAC) definition and diagnostic criteria for hepatorenal syndrome

***1996 Criteria *** [39]

**Major Criteria**
• Chronic or acute liver disease with advanced hepatic failure and portal hypertension.
• Serum creatinine > 1.5 mg/dL or 24-h creatinine clearance of < 40 mL/min.
• Absence of shock, ongoing bacterial infection, and current or recent treatment with nephrotoxic drugs. Absence of gastrointestinal fluid losses (repeated vomiting or intense diarrhea) or renal fluid losses
• No sustained improvement in renal function defined as a decrease in serum creatinine to < 1.5 mg/dL or increase in creatinine clearance to 40 mL/min or more following diuretic withdrawal and expansion of plasma volume with 1.5 L of isotonic saline.
• Proteinuria < 500 mg/dL and no ultrasonographic evidence of obstructive uropathy or parenchymal renal disease.
**Minor Criteria**
• Urine volume < 500 mL/d
• Urine sodium < 10 mEq/L
• Urine osmolality > plasma osmolality
• Urine red blood cells < 50 per high power field
***2007 Criteria *** [40]
• Cirrhosis with ascites
• Serum creatinine > 1.5 mg/dL
• No improvement of serum creatinine (decrease to a level ≤ 1.5 mg/dL) after at least two days of diuretic withdrawal and volume expansion with albumin. The recommended dose of albumin is 1 g/kg of body weight per day up to a maximum of 100 g/day
• Absence of shock
• No current or recent treatment with nephrotoxic drugs
• Absence of parenchymal kidney disease as indicated by proteinuria > 500 mg/day, microhematuria (> 50 red blood cells per high power field), and/or abnormal renal ultrasonography

**Table 4:** The Acute Dialysis Quality Initiative (ADQI) criteria for the definition and classification of acute kidney injury (modified RIFLE criteria) [41,43]

AKI Stage	Serum creatinine criteria	Urine output criteria
1 (Risk)	Increase Scr ≥ 0.3 mg/dL within 48 hours or an increase 150 - 200% (1.5- to 2-fold) from baseline	< 0.5 ml/kg/hour for > 6 hours
2 (Injury)	Increase Scr 200% to 299% (≥ 2- to 3-fold) from baseline	< 0.5 ml/kg/hour for > 12 hours
3 (Failure)	Increase Scr ≥ 300% (≥ 3-fold) from baseline or Scr ≥ 4.0 mg/dL with an acute increase of ≥ 0.5 mg/dL or initiation of renal replacement therapy	< 0.3 ml/kg/hour for 24 hours or anuria for 12 hours

Scr = serum creatinine

**Figure 1:**
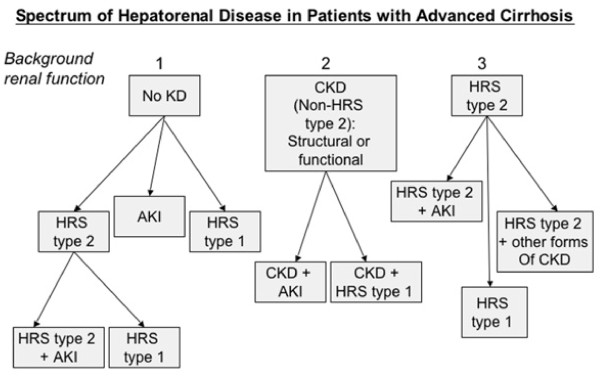
**Classification of hepatorenal disorder (HRD).** Spectrum of hepatorenal disorders in patients with advanced cirrhosis. AKI = acute kidney injury; CKD = chronic kidney disease; KD = kidney disease; HRS = hepatorenal syndrome. (With permission)^48^

In critically ill patients with cirrhosis, AKI defined by RIFLE criteria has been shown to be a predictor of hospital survival [44-46]. The final consensus of the workgroup was to apply the RIFLE criteria to define AKI in patients with cirrhosis irrespective of whether the cause of the acute deterioration in renal function was related to a functional or structural disorder (Table 5) [47]. This will certainly identify many patients with AKI with normal S_Cr _but low GFR and allow us to extend treatment to patients at a lesser level of severity. In addition, the overall consensus was to also propose that the term hepatorenal disorders (HRD) be used to describe all patients with advanced cirrhosis and concurrent kidney dysfunction (Figure 1). HRD is thus defined as any form of kidney disease occurring in patients with cirrhosis, be it functional or structural in nature. Such a definition will allow cirrhotic patients with renal dysfunction to be properly classified, thereby allowing appropriate studies to be conducted to define their prognosis and to devise treatment options.

**Table 5:** Proposed diagnostic criteria of kidney dysfunction in cirrhosis [47]

Diagnosis	Definition
**Acute Kidney Injury**	• A rise in Scr ≥ 50% from baseline, or a rise Scr > 0.3 mg/dL • Type-1 HRS is a specific form of acute kidney injury
**Chronic Kidney Disease**	• GFR < 60 ml/min for > 3 month calculated using MDRD-6 formula
**Acute on Chronic Kidney Disease**	• Rise in Scr ≥ 50% from baseline or a rise of Scr > 0.3 mg/dL in a patient with cirrhosis whose GFR is < 60 ml/min for > 3 month calculated using MDRD-6 formula

GFR, glomerular filtration rate; HRS, hepatorenal syndrome; Scr, serum creatinine. Both the acute deterioration in renal function and the background chronic renal dysfunction can be functional or structural in nature. MDRD-6: GFR = 170 × Scr (mg/dL)^-0.999 ^x age^-0.176 ^x 1.180 (if black) × 0.762 (if female) × serum urea nitrogen^-0.170 ^× albumin^0.138^

#### 2. Classify CKD in the setting of cirrhosis according to Kidney Disease Outcomes Quality Initiatives (K/DOQI) (Not Graded)

**Rationale: **As mentioned above, estimation of GFR in cirrhosis is problematic; therefore, the application of the definition of CKD in cirrhosis is challenging. The Work Group accepted the definition of CKD, as set out by the practice guidelines from the K/DOQI Work Group [48], of an eGFR < 60 mL/min/1.73 m^2 ^calculated using the MDRD-6 formula [29] for more than three months for patients with cirrhosis (Table 5) [47]. Patients with type-2 HRS who meet these criteria should be considered as having CKD.

#### 3. Acute on CKD in cirrhosis is defined as a rise in S_Cr _≥ 0.3 mg/dL in less than 48 hours or an increase in S_Cr _≥ 50% from baseline, or in a patient with cirrhosis whose baseline GFR has been < 60 ml/min calculated with the MDRD-6 formula for more than three months (Not Graded)

**Rationale: **It is important to recognize that AKI may also occur in patients with preexisting HRD. The group recognizes that acute on CKD does occur in cirrhosis and has defined it using the RIFLE criteria for AKI and K/DOQI guidelines for CKD (Table 5) [47].

Recommendations for future research:

• Multicenter, prospective, epidemiologic (observational) studies to:

- Investigate the incidence, prevalence, basic demographics and outcomes of patients with HRD

- Validate new diagnostic criteria of AKI, CKD and acute on CKD for the cirrhotic population

- Determine whether the development of type-1 HRS in a patient with pre-existing type-2 HRS is the same as patients with pre-existing CKD

- Investigate the use of biomarkers to differentiate between type-1 HRS and other forms of AKI in patients with cirrhosis.

### III. Pharmacologic treatment of HRS

#### 1. We suggest using hemodynamic monitoring, when possible, to help with the management of fluid balance in patients with HRS (2D)

**Rationale: **The key management strategy for patients admitted with cirrhosis is to avoid the development of HRS by preventing relative renal hypoperfusion, maintaining an effective circulating volume and renal perfusion pressure. Assessment of intravascular volume in patients with HRS, however, is challenging. Traditional methods based on clinical examination and static measurements of right atrial and pulmonary artery pressures are of questionable accuracy in predicting volume responsiveness and should be used with caution [49-53]. Goal directed therapy with 20% albumin increased central blood volume and cardiac index, without subsequent changes in central venous pressure [54] as any fluid bolus which initially expands the intravascular space, will subsequently expand the 'third space'. Functional hemodynamic monitoring, using continuous central venous pressures or indirect measurements of cardiac indices, should be used when possible to assess the short term response to a fluid volume bolus [55].

Although pulse pressure variation (PPV) derived from analysis of the arterial waveform and the stroke volume variation (SVV) derived from pulse contour analysis are predictive of fluid responsiveness in patients receiving volume-controlled mechanical ventilation [51], they are not as reliable in septic patients and those on pressure-support ventilation [53]. Serial echocardiography can be used to assess changes in intravascular volume by measuring inferior vena caval diameter, right ventricular end-diastolic volume index, left ventricular end-diastolic area index, and the global end-diastolic volume index, but generally these are not as accurate as using PPV/SVV, are operator dependent and have not been evaluated in patients with cirrhosis [56,57].

#### 2. We recommend that patients with type-1 HRS be optimally resuscitated with albumin (initially 1 g of albumin/kg for two days, up to a maximum of 100 g/day, followed by 20 to 40 g/day) in combination with a vasoconstrictor (1A), preferentially terlipressin (2C)

**Figure 2:**
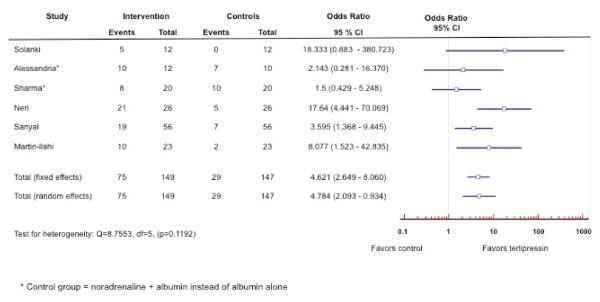
**Forest plot of meta-analysis on terlipressin plus albumin for patients with hepatorenal syndrome.** The outcome measure is reversal of hepatorenal syndrome.

**Figure 3:**
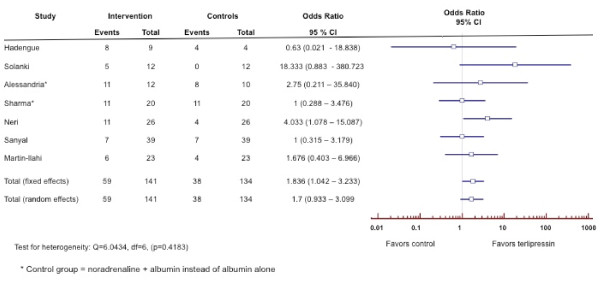
**Forest plot of meta-analysis on terlipressin plus albumin for patients with hepatorenal syndrome.** The outcome measure is survival.

**Rationale: **The most effective method for treatment of type-1 HRS currently available is the administration of systemic vasoconstrictor drugs (Table 6) in order to reduce the marked vasodilatation in the splanchnic and systemic circulations thereby improving the associated impaired circulatory function [58,59]. Although randomized controlled trials [60-77] and meta-analysis [78,79] of the combination of terlipressin and albumin have been shown to reverse HRS type 1 and improve renal function in patients with type-1 HRS (Figure 2), data regarding survival benefit has been limited although it may allow survival to transplantation (Figure 3). Therapy should be discontinued after four days in non-responders and only continued thereafter in partial responders (S_Cr _improves, but does not decrease to < 1.5 mg/dL). As terlipressin can cause organ ischemia, it is contraindicated in patients with ischemic heart disease, peripheral vascular disease and/or cerebrovascular disease and all patients should be monitored closely for cardiac arrhythmias or signs of splanchnic or digital ischemia. If terlipressin is unavailable, alternative vasoconstrictors such as norepinephrine [69,77,80], vasopressin [81] or a combination of octreotide and midodrine [82-87], together with albumin should be considered. Currently there are no randomized trials showing superiority of terlipressin in comparison to other vasoconstrictors.

**Table 6:** Vasoconstrictor drugs for the treatment of hepatorenal syndrome

Drug	Dose
Terlipressin [60-78]	0.5 to 2.0 mg intravenously every 4 to 6 hours; with stepwise dose increments every few days if there is no improvement in serum creatinine, up to a maximum dose of 12 mg/day as long as there are no side effects. Maximal treatment 14 days
Vasopressin [81]	0.01 U/min to 0.8 U/min (continuous infusion). Titrate to achieve a 10 mm Hg increase in MAP from baseline or MAP > 70 mmHg
Noradrenaline [69,77,80]	0.5 to 3.0 mg/hour (continuous infusion). Titrate to achieve a 10 mmHg increase in MAP
Midodrine + Octreotide [82-87]	Midodrine: 7.5 to 12.5 mg orally three times. Titrate to achieve a 15 mm Hg increase in MAP from baseline Octreotide:100 to 200 μg subcutaneously three times daily or 25 μg bolus, followed by intravenous infusion of 25 μg/hour

MAP: mean arterial pressure

Recommendations for future research:

• Comparative randomized controlled trials of vasoconstrictors are required to determine the merits of vasopressin analogues, specifically terlipressin, against alpha-adrenergic agents in patients with type-1 HRS.

• Randomized controlled trials of vasoconstrictors in patients with type-2 HRS.

### IV. Device management of hepatorenal syndrome

#### 1. We recommend withholding renal replacement therapy (RRT) in patients with decompensation of cirrhosis who are not candidates for liver transplantation (1D)

**Rationale***: *RRT improves short-term survival in severe AKI and can be helpful in bridging patients to transplant or treating patients who have acute but reversible decompensation [88]. Although there is a preference for continuous renal replacement therapy (CRRT) over intermittent hemodialysis in hemodynamically unstable patients [89-93], analysis of the currently published studies does not allow evidence-based guidelines for the selection of RRT modality for the treatment of AKI in the setting of HRS. CRRT use may be advantageous in the management of HRS patients with AKI who are hemodynamically unstable or those patients at risk of elevated intracranial pressure such as patients with acute fulminant liver failure or acute on chronic liver failure [94,95]. However, prognosis in type-1 HRS is very poor and RRT should be avoided in these patients unless there is either an acute reversible component or a plan for liver transplantation.

#### 2. We suggest that artificial liver support therapies for HRS be limited to research protocols (2D)

**Figure 4:**
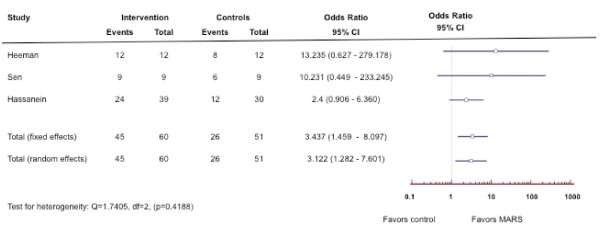
**Forest plot of meta-analysis on Molecular Adsorbent Recirculating System (MARS) for patients with hepatorenal syndrome. **The outcome measure is improvement of hepatic encephalopathy.

**Figure 5:**
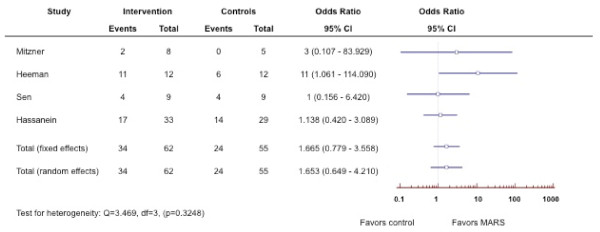
**Forest plot of meta-analysis on Molecular Adsorbent Recirculating System (MARS) for patients with hepatorenal syndrome. **The outcome measure is survival.

**Rationale: **Extracorporeal support systems in liver disease can be divided into two broad categories: non-cell [96-131] and cell-based systems (Table 7) [132-134]. Non-cell based systems do not incorporate tissue and provide only detoxification functions using membranes and adsorbents. These newer developing therapies are expensive and have not as yet demonstrated a survival benefit in patients with type-1 HRS despite improvements in neurological function and coagulation indices. Molecular Adsorbent Recirculating System (MARS) is now commercially available in the US and Europe. Although MARS therapy has not demonstrated a definite survival benefit in patients with liver disease, it has been shown to improve hepatic encephalopathy (Figures 4, 5) [125-131]. Cell-based systems aim to provide the excretory, synthetic, and metabolic functions of the liver using living liver cells. In addition to detoxification, cell-based systems can also provide some synthetic and regulatory functions; however, they have mainly been tested in single-center phase I and II trials, and none have yet received US Food and Drug Administration (FDA) approval.

**Table 7:** Extracorporeal liver support system

	Technique
**Artificial (Non-cell based)**	
Hemoperfusion [96-101]	Removal of protein-bound toxins by circulating blood over a sorbent material
Hemodiabsorption [102-105]	Hybrid process in which blood is passed through a hemodialyzer containing a suspension of sorbent material, such as charcoal or resin, in the extracapillary space
Plasma Exchange [106-109]	Exchange of plasma volume
Plasmapheresis [110]	Plasma is separated from the cellular blood components and replaced with normal plasma constituents, allowing the removal of circulating toxins and waste products.
Plasma Filtration [111-117]	Removes a specific plasma fraction containing substances within a specific molecular weight.
Albumin dialysis	Albumin containing dialysate using an anion exchange resin and active charcoal adsorption allowing albumin-bound toxins in the blood to cross the membrane and bind to the albumin. Water soluble toxins are dialyzed from the albumin circuit by a standard hemodialysis or continuous renal replacement therapy (CRRT) machine.
• Single Pass Albumin Dialysis (SPAD) 118-121]	
• Prometheus [122-124]	
• Molecular Adsorbent Recirculating System (MARS) [125-131]	
	
**Bioartificial (Cell-based) **[132-134]	
Porcine	
• HepatAssist • Bioartificial Liver Support System (BLSS) • Modular Extracorporeal Liver Support (MELS) • Hybrid-Bioartificial Liver (HBAL) • Radial Flow Bioreactor (RFB) • TECA-Hybrid Artificial Liver Support System • AMC-Bioartificial Liver	
Human	
• Extracorporeal Liver Assist Device (ELAD)	

Recommendations for future research:

• Further observational studies and ultimately randomized controlled trials directed to the identification of the appropriate indications, timing of intervention, and cost effectiveness of supportive detoxification therapies. Consequently, the most appropriate outcome for studies would be short-term survival as a bridge to transplantation.

### V. Interventional and surgical management of HRS

#### 1. We recommend use of a transjugular intrahepatic portosystemic shunt (TIPS) as a treatment option for patients with type-2 HRS with refractory ascites who require large-volume paracentesis (1C)

**Rationale***: *Very few studies have assessed the role of TIPS in patients with HRS (Table 8) [135-138]. In patients with type-2 HRS, TIPS has been shown to improve refractory ascites and improve renal function without improvement in survival [138]. However, new hepatic encephalopathy, deterioration of previous hepatic encephalopathy [136] or mild and transient deterioration of liver function tests [137] has been reported following TIPS. In patients with type-1 HRS, TIPS may improve renal function and survival [136,137]; however, there is insufficient data to support the use of TIPS as a first-line treatment of patients with type-1 HRS. TIPS is not recommended in patients with severe liver failure defined as serum bilirubin > 5 mg/dl, INR > 2 or Child-Pugh score > 11, hepatic encephalopathy or severe cardiopulmonary diseases.

**Table 8:** Clinical trials on transjugular intrahepatic portosystemic shunt (TIPS) in patients with hepatorenal syndrome

Author	Year	N	Type of Study	Study Population (n)	Pre-TIPS Cr	Post-TIPS cr	Survival
Brensing [135]	1997	16	Prospective uncontrolled	HRS Type 1 & 2	2.57 ± 1.59 mg/dl	1.18 ± 0.59 mg/dl	56%
Guevara [136]	1998	7	Prospective uncontrolled	HRS Type 1	5.0 ± 0.8 mg/dl	1.8 ± 0.4 mg/dl	28% Mean survival: 140 ± 68 days
Brensing [137]	2000	41	Prospective	Non-TIPS (*n *= 10)HRS Type 1 (*n *= 7) HRS Type 2 (*n *= 3)	2.3 ± 1.7 mg/dl	1.5 ± 1.2 mg/dl	Mean Survival: TIPS: 92 ± 16 weeks Non TIPS: 12 ± 8.5 weeks
				TIPS (*n *= 31) HRS Type 1 (*n *= 14) HRS Type 2 (*n *= 17)			3 month survival TIPS: 81% Non TIPS:10%
Testino [138]	2003	18	Prospective uncontrolled	HRS Type 2 (*n *= 18)	1.9 ± 0.5 mg/dl	0.9 ± 0.3 mg/dl	67%: Transplanted

#### 2. We suggest liver transplantation alone for candidates with type-1 HRS for less than four weeks and simultaneous liver-kidney (SLK) for those at risk for non-recovery of renal function (2D)

**Rationale: **As the waiting time for liver transplantation has increased, the incidence of pre-transplant renal dysfunction and RRT has also increased. In patients with renal dysfunction at the time of liver transplantation (LT), it is important to know whether renal function will improve, stabilize or continue to progress following transplantation. However, the level and duration of renal dysfunction, including RRT, beyond which renal recovery is not possible following LT alone is unknown. Several investigators have studied the impact of pre-transplant AKI on post transplant outcomes; however, the small study sample size, retrospective design, reporting bias, and variation in definitions used to define renal dysfunction hinders comparison between these studies [139-145].

**Figure 6:**
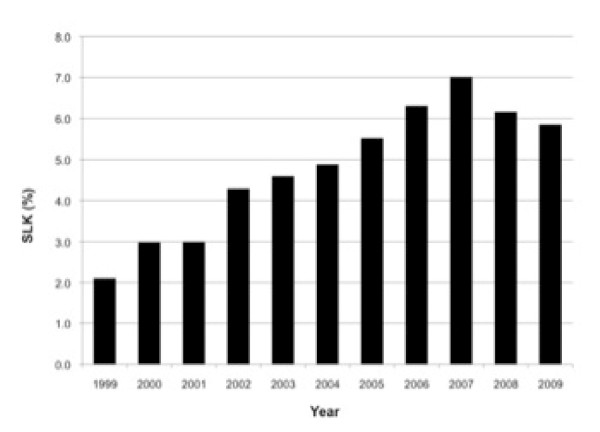
Percent of Adult Simultaneous Liver-Kidney (SLK) Transplant Amongst all Cadaveric Liver Transplant Recipients (1999-2009).

Currently there are no standard criteria for the evaluation, selection and/or allocation of a kidney at the time of LT. Despite an increase in the rate of SLK transplantation immediately following the implementation of the MELD allocation system in March 2002 (Figure 6), recent Organ Procurement and Transplantation Network (OPTN) data suggest a decrease since 2007 which may be due to consensus guidelines published at that time [146,147]. Based on the published consensus guidelines, the OPTN Liver and Intestine Committee and Kidney Committee recently developed proposed listing criteria for SLK candidate selection and allocation (Policy 3.5.10) (Table 9).

**Table 9:** Criteria for simultaneous liver-kidney transplant candidates as proposed by the UNOS Kidney Transplantation Committee and the Liver and Intestinal Organ Transplantation Committee (UNOS Policy 3)

a. Chronic kidney disease (CKD) requiring dialysis with documentation of the CMS form 2728^a^
b. Chronic kidney disease (GFR < = 30 ml/min by MDRD6 or iothalamate measurement and proteinuria > 3 gms/day with 24 hour protein measurement or urine protein/creatinine ratio > 3 not requiring dialysis
c. Sustained acute kidney injury requiring dialysis for 6 weeks or more (defined as dialysis at least twice per week for 6 consecutive weeks)
d. Sustained acute kidney injury (GFR < = 25 ml/min for 6 weeks or more by MDRD6 or direct measurement) not requiring dialysis
e. Sustained acute kidney injury: Patients may also qualify for SLK listing with a combination of time in categories (c) and (d) above for a total of six weeks (or example, patients with a GFR < 25 ml/min for 3 weeks followed by dialysis for 3 weeks).
f. Metabolic Disease

CKD, chronic kidney disease; CMS, Center for Medicare & Medicaid Services; GFR, glomerular filtration rate; MDRD-6, Modification of Diet in Renal Disease formula calculated using 6 variables of serum creatinine, serum urea, serum albumin, age, gender and whether the patient is African-American or not.

^a^CMS form 2728: Form required by Medicare & Medicaid stating that a dialysis patient has end-stage renal disease with no chance of renal recovery

The duration of pre-liver transplant kidney dysfunction or dialysis that is amenable to recovery is not known. Retrospective studies from single centers have shown the importance of the duration of > 12 weeks of S_Cr _≥ 1.5 mg/dL and dialysis ≤ 4 weeks pre-transplant on post transplant renal outcomes [140,143-145]. However, the renal outcome of patients dialyzed for > 4 weeks is unknown as many of these patients frequently undergo SLK. Timing of dialysis is related to numerous factors and is a complex process. Since a threshold of dialysis duration that is sufficiently predictive of renal recovery has not been established, and also because initiation of dialysis is physician/center dependent, dialysis duration as a criterion for SLK should be used with caution in patients who do not have end-stage renal disease. The decision for SLK versus LT alone should be undertaken with consideration of duration of HRS, AKI and CKD and risk factors for progression of CKD present at the time of liver transplant such as hypertension, diabetes and obesity. Currently there are several pitfalls with the SLK guidelines such as definition of AKI, GFR determination, timing of initiation of dialysis and duration of dialysis. To that end, the ADQI group unanimously agreed that using the existing literature is not sufficient to allow guidelines or criteria to be set.

Recommendations for future research:

• Multicenter, prospective, observational studies in patients undergoing SLK to determine:

- The predictive value of RIFLE classifications pre-transplant on post liver transplant outcomes

- Which RIFLE class and for what duration of AKI pre-transplant is associated with rates of renal recovery that warrant SLK

- What is the recovery rate of native kidney function in patients with pre-transplant type-1 or 2 HRS

• Epidemiologic studies that document long-term patient and renal outcomes and the prognostic factors for these outcomes in patients with HRS who receive LT alone, who recover residual renal function, remain on dialysis and those subsequently receiving renal transplantation.

## Conclusions

Since first defined in 1996 by the IAC, there has been substantial progress towards understanding the pathogenesis and natural history of HRS. However, there remains a significant deficiency in our knowledge regarding its management. The IAC has set out clear diagnostic criteria for both acute and chronic forms of HRS, but has not delineated guidelines for the diagnosis of other forms of renal impairment in cirrhosis, be they acute or chronic. Well-accepted definitions and staging systems for CKD and AKI exist, but have not been consistently applied to patients with advanced liver disease. Indeed, it must be understood that although our recommendations are based, to the best extent possible, on data, there are insufficient data to guide many important decisions. In addition, although our Work Group included a wide range of specialists from around the world, we do recognize that there exists regional heterogeneity in practice. As a result, our findings should be considered a 'first step' in the process of standardization of the definition of AKI in patients with cirrhosis. Once agreed, these new diagnostic criteria of AKI for cirrhosis will need to be validated and the threshold for the diagnosis of type-1 HRS may need to be revised to a lower target value to allow patients with a lesser degree of S_Cr _rise to receive prompt appropriate treatment. In addition, criteria for SLK may need to be revisited in lieu of the new classification and to determine whether the new classification and definition will improve patient outcomes. One clear conclusion of this ADQI meeting is that multicenter, prospective, long-term outcome studies in patients with advanced cirrhosis and HRS are urgently needed.

## Key messages

• The ADQI Work Group recommends incorporation of the modified RIFLE criteria to define AKI in patients with cirrhosis irrespective of whether the cause of the acute deterioration in renal function is related to a functional or structural discorder.

• Regarding the current state of combined liver-kidney transplantation in patients with cirrhosis and kidney disease, including HRS, the ADQI group unanimously agreed that using the existing literature is not sufficient to allow guidelines or criteria to be set.

• Multicenter prospective, long-term outcome studies in patients with advanced cirrhosis and HRS are urgently needed.

## Abbreviations

ADQI: Acute Dialysis Quality Initiative; AKI: acute kidney injury; CKD: chronic kidney disease; CKD-EPI: Chronic Kidney Disease Epidemiology Collaboration; CRRT: continuous renal replacement therapy; eGFR: estimated glomerular filtration rate; FDA: Food and Drug Administration; GFR: glomerular filtration rate; HRD: hepatorenal disorders; HRS: hepatorenal syndrome; IAC: International Ascites Club; IL: interleukin; KIM-1: kidney-injury molecule 1; K/DOQI: Kidney Disease Outcomes Quality Initiatives; LT: liver transplantation; MARS: molecular adsorbent recirculating system; MDRD: Modified Diet in Renal Disease; MELD: Model for End-Stage Liver Disease; NGAL: neurtrophil gelatinase-associated lipocalin; OPTN: Organ Procurement and Transplantation Network; PPV: pulse pressure variation; RIFLE: risk: injury: failure: loss: end-stage; RRT: renal replacement therapy; Scr: serum creatinine; SLK: simultaneous liver-kidney; SVV: stroke volume variation; TIPS: transjugular intrahepatic portosystemic shunt.

## Competing interests

ADQI is supported by a group of sponsors and no funding is accepted for the development of specific guidelines and every effort is made by to avoid any actual or perceived conflicts of interest that may arise as a result of an outside relationship of a member of the ADQI Work Group. Prior to the meeting, all members of the Work Group are required to complete, sign and submit a disclosure and attestation form showing all their relationships that might be actual or perceived conflicts of interest. None of the companies that provided financial support for this meeting were involved in any way in the organization of the meeting, the decision regarding invited faculty, the design and contents of the meeting, the collection, management, analysis, and interpretation of the data, and preparation, review, or approval of the manuscript. MKN has served as an advisor for Ikaria and has also served as a speaker for Gambro. JAK has served as a consultant and has received grant support from Gambro and Baxter. AT, JC and NG have served as speakers for Gambro. CD has served as an advisor for Ikaria. The other 13 members of the Work Group reported no relevant financial relationships.

## Authors' contributions

MKN, JAK and YSG were the main organizers of this meeting and helped to draft the manuscript. AD, CD, NP, RB, FW and AT were the facilitators for each Work Group and participated in drafting the sections that were related to their Work Group. All authors read and approved the final manuscript.
